# Genomic arrangement of salinity tolerance QTLs in salmonids: A comparative analysis of Atlantic salmon (*Salmo salar*) with Arctic charr (*Salvelinus alpinus*) and rainbow trout (*Oncorhynchus mykiss*)

**DOI:** 10.1186/1471-2164-13-420

**Published:** 2012-08-24

**Authors:** Joseph D Norman, Mike Robinson, Brian Glebe, Moira M Ferguson, Roy G Danzmann

**Affiliations:** 1 Department of Integrative Biology, University of Guelph, Guelph, ON N1G 2W1, Canada; 2 Golder Associates Ltd, 102, 2535 – 3rd Avenue S.E, Calgary, AB T2A 7W5, Canada; 3 Department of Fisheries and Oceans, St. Andrews Biological Station, St. Andrews, New Brunswick, E5B 2L9, Canada

**Keywords:** Atlantic salmon, Arctic charr, Rainbow trout, Salinity tolerance, Genome rearrangements, Whole-genome duplications, Genome synteny, Candidate genes, Teleost fishes

## Abstract

**Background:**

Quantitative trait locus (QTL) studies show that variation in salinity tolerance in Arctic charr and rainbow trout has a genetic basis, even though both these species have low to moderate salinity tolerance capacities. QTL were observed to localize to homologous linkage group segments within putative chromosomal regions possessing multiple candidate genes. We compared salinity tolerance QTL in rainbow trout and Arctic charr to those detected in a higher salinity tolerant species, Atlantic salmon. The highly derived karyotype of Atlantic salmon allows for the assessment of whether disparity in salinity tolerance in salmonids is associated with differences in genetic architecture. To facilitate these comparisons, we examined the genomic synteny patterns of key candidate genes in the other model teleost fishes that have experienced three whole-genome duplication (3R) events which preceded a fourth (4R) whole genome duplication event common to all salmonid species.

**Results:**

Nine linkage groups contained chromosome-wide significant QTL (AS-2, -4p, -4q, -5, -9, -12p, -12q, -14q -17q, -22, and −23), while a single genome-wide significant QTL was located on AS-4q. Salmonid genomes shared the greatest marker homology with the genome of three-spined stickleback. All linkage group arms in Atlantic salmon were syntenic with at least one stickleback chromosome, while 18 arms had multiple affinities. Arm fusions in Atlantic salmon were often between multiple regions bearing salinity tolerance QTL. Nine linkage groups in Arctic charr and six linkage group arms in rainbow trout currently have no synteny alignments with stickleback chromosomes, while eight rainbow trout linkage group arms were syntenic with multiple stickleback chromosomes. Rearrangements in the stickleback lineage involving fusions of ancestral arm segments could account for the 21 chromosome pairs observed in the stickleback karyotype.

**Conclusions:**

Salinity tolerance in salmonids from three genera is to some extent controlled by the same loci. Synteny between QTL in salmonids and candidate genes in stickleback suggests genetic variation at candidate gene loci could affect salinity tolerance in all three salmonids investigated. Candidate genes often occur in pairs on chromosomes, and synteny patterns indicate these pairs are generally conserved in 2R, 3R, and 4R genomes. Synteny maps also suggest that the Atlantic salmon genome contains three larger syntenic combinations of candidate genes that are not evident in any of the other 2R, 3R, or 4R genomes examined. These larger synteny tracts appear to have resulted from ancestral arm fusions that occurred in the Atlantic salmon ancestor. We hypothesize that the superior hypo-osmoregulatory efficiency that is characteristic of Atlantic salmon may be related to these clusters.

## Background

The life history of anadromous salmonids entails movements between freshwater and seawater environments. To cope with the physiological demands associated with such behaviour, salmonids and other teleosts have evolved the capacity to change their ionoregulatory condition by altering states of ion uptake (hyper-osmoregulation) and excretion (hypo-osmoregulation). In teleosts, mitochondria-rich cells are located in the epithelial layer of fish gill tissue and play a major role in hypo-osmoregulation. The molecular mechanisms within and around mitochondria-rich cells function together to facilitate the removal of Na^+^ and Cl^-^ from blood plasma and are an integral part of the mitochondria-rich cell model [1]. Active exchange of cytoplasmic Na^+^ with intercellular K^+^ by Na^+^/K^+^-ATPase pumps maintains an electrochemical gradient sufficient to move intercellular Cl^-^ across the basolateral membrane and into the cell via Na^+^/K^+^/2Cl^-^ (NKCC) cotransporters. Cl^-^ exits the cell across the apical membrane through embedded cystic fibrosis transmembrane conductance regulator-like anion channels (CFTR). Intercellular Na^+^ is secreted through leaky cation-selective paracellular pores between accessory cells and mitochondria-rich cells [1,2], where variation in permeability rates are thought to be a function of claudin isoform type [3,4].

Several genes that are not part of the current mitochondria-rich cell model are also involved in the hypo-osmoregulatory process. Insulin-like growth factor 2 (IGF2) transcription is positively correlated with elevated salinity tolerance [5]. Growth hormone (GH) levels [6] also regulate this balance, as illustrated by GH injections that correlate with a rapid increase in salinity tolerance capacity [7]. Such short-term effects could be related to changes in gill tissue cell structure, as GH has been connected with seawater-induced mitochondria-rich cell hyperplasia and hypertrophy [8,9]. Moreover, Atlantic salmon smolts (i.e., fish prepared for seawater) have larger relative gill weights compared to non-smolts (i.e., fish prepared for freshwater) of equal size [10], which suggests that smoltification-induced changes in cell structure may be reflected by changes in gill weight. The localization of salinity tolerance QTL in multiple species to linkage groups where GH (i.e., on RT-9q & AC-20) and IGF2 are mapped (i.e., on RT-27 & AC-4/19) [11-15] is consistent with the hypothesis that allelic variation at the loci encoding these hormones exerts significant affects on hypo-osmoregulatory capacity. Unsurprisingly, growth hormone receptor (GHR), which modulates tissue-specific activity of GH [16], is upregulated concurrently with elevated salinity and the onset of smoltification in rainbow trout and Atlantic salmon, respectively [17,18]. Collagen type I alpha I (COL1A1), secreted protein acidic and rich in cysteine (SPARC) [19], and calcium-sensing receptors (CaSR) [20] are also upregulated in Atlantic salmon smolts. COL1A1 appears to be involved in the composition of the extracellular matrix and arch formation of fish gills [19], while SPARC has been associated with tissue remodelling. By binding to structural proteins such as collagen type I, SPARC regulates cellular interactions with the extracellular matrix [21]. Lastly, calcium-sensing receptors (CaSR) are thought to act as osmosensors by sending regulatory signals in response to elevated plasma ion concentrations [20].

Within the Salmonidae family there is wide variation in both anadromy and salinity tolerance capacity among species [22-27]. Sea-run Atlantic salmon (*Salmo salar*) are iteroparous and have high saltwater tolerance, spending multiple years at sea between river spawning migrations. At the other extreme, anadromy in Arctic charr (*Salvelinus alpinus*) involves shorter periods of seawater residency (i.e., a few months), which is accompanied by a relatively low capacity for hypo-osmoregulation [9,26,28]. Both of these species also show wide intraspecific variation in salinity tolerance capacity.

While in seawater, different Atlantic salmon families show large differences in blood plasma osmolality concentrations [24], while wide disparity in seawater-induced mortality [29] has been observed in different strains of Arctic charr, as have family-based differences in Na^+^/K^+^-ATPase activity levels and blood plasma osmolality concentrations [15]. Such studies suggest that inter- and intraspecific variation in salinity tolerance capacity is affected by genetic variation. In fact, recent research shows that quantitative trait loci (QTL) for various salinity tolerance performance indicator traits localize to homologous linkage groups in rainbow trout [11] and Arctic charr [15]. These QTL localize to linkage groups that are predicted to contain genes for the primary mechanisms from the mitochondria-rich cell model (e.g., ATP1α1b, NKCC, and CFTR) [15].

The extent that the genetic basis of salinity tolerance is conserved across Salmonidae species is unclear. Well established chromosome homologies among Arctic charr and rainbow trout [30,31] have facilitated assessments of the conservation of salinity tolerance QTL. Several tentative QTL homologies have been identified, but differences in experimental design and genetic map resolution have made comparisons difficult. Assessment of the salinity tolerance QTL positions in Atlantic salmon would contribute knowledge on the conserved homologous QTL genomic locations influencing this trait in salmonids.

The evolutionary lineage of modern salmonids is unique in that it is punctuated by four whole-genome duplication events. Two whole-genome duplication events have occurred in all vertebrate lineages (i.e., 1R, 2R) [32]. Doubly conserved synteny blocks among *Tetraodon nigroviridis* and *Homo sapiens*[33] show that a third whole-genome duplication occurred in fishes (i.e., 3R). In modern salmonids, residual tetrasomic inheritance, multivalent formations, and *Hox* gene duplication patterns suggest that a fourth whole-genome duplication occurred in the salmonid ancestor some 25-100 million years ago (i.e., 4R) [34-36]. Evidence suggests that the 4R duplication may have had ramifications for the evolution of salinity tolerance, for in Arctic charr there are multiple instances where trait-specific QTL localize to homeologous linkage groups (e.g., predicted duplicates ATP1α1b loci overlap with QTL detected on AC-12 and -27) [15,30,37,38]. Such patterns would be expected if duplicated loci were subfunctionalized or if a redundant locus was neofunctionalized, contrary to the usual fate of duplicated loci (pseudogenization) [39]. In addition, duplicate gene function may simply be retained because of positive selective advantage. On the other hand, patterns suggestive of QTL homeology would be indistinguishable from patterns resulting from QTL linked to non-paralogous loci, if such loci were tightly linked in the salmonid ancestor prior to the 4R duplication event and have since been conserved on only reciprocal homeologues in extant salmonids (see doubly conserved synteny blocks in [33]). Interestingly, the latter scenario is consistent with observations that suggest some salinity tolerance candidate genes occur in clusters within the same linkage group region [15], and could singly or in combination contribute to the apparent conserved QTL homeologies that were identified.

Disparity in genomic structure among Atlantic salmon (2n = 54-58; NF = 72–74), Arctic charr (2n = 78; NF = 100) and rainbow trout (2n = 58-64; NF = 100-104) [40,41] could have lead to differences in the arrangement of genes related to salinity tolerance. The non-randomness of gene order [42] in conjunction with the co-expression of gene clusters in eukaryotes [43] implies that gene arrangement is important in the evolution of phenotypes. More specifically, it suggests that genes in close proximity are more likely to be involved in the same biochemical pathway [44], and that disruptions in these regions could conceivably affect development of the respective phenotype. The availability of the reconstructed 2R proto-Actinopterygian ancestral karyotype and the sequenced genomes of multiple 3R teleost species provide an opportunity to examine the extent that genomic rearrangements may have affected the relative positions of salinity tolerance candidate genes in 3R and 4R genomes. The approximate positions of candidate genes in 4R genomes can be predicted using knowledge of their precise positions in 3R genomes in conjunction with synteny patterns evident among 3R and 4R species. Comparisons with the positions of salinity tolerance QTL would then provide an opportunity to assess whether disparity in salinity tolerance capacity could be correlated with differences in genomic structure.

The characterization of salinity tolerance QTL in Atlantic salmon and subsequent comparisons with Arctic charr and rainbow trout will allow us to determine which QTL are conserved across species using information from three different genera (Salmo, Salvelinus, and Oncorhynchus). Comparative genomics approaches will also allow us to infer if genomic rearrangements have affected the relative positions of salinity tolerance candidate genes in the genomes of salmonids. Using two out-bred Atlantic salmon families, we examined the genetic architecture of salinity tolerance performance traits after exposure to seawater in a controlled environment designed to simulate natural conditions. We addressed the following questions: (1) Does genetic variation have a significant effect on salinity tolerance performance traits in Atlantic salmon? (2) Do salinity tolerance QTL share homeologous affinities? (3) Do QTL share homologous affinities with QTL in other salmonids? (4) Do QTL localize to linkage groups that contain or are predicted to contain candidate genes? (5) Is the relative arrangement of candidate genes different among 2R, 3R, and 4R species? (6) Is disparity in salinity tolerance capacity among salmonid species correlated with variation in genomic structure?

## Results

### Genetic maps

From the parents of two full-sib families (i.e., family numbers 7 and 9) four sex-specific maps were created (see Additional file 1, Additional file 2, Additional file 3, and Additional file 4). Eight to seventeen linkage groups per family were comprised of a minimum of two markers. Twenty-three of a possible maximum of 29 [41] chromosomes were represented by linkage groups from all parents combined. Linkage group 18 was represented by a single unlinked marker. Five linkage groups were not included in this study due to an absence of marker polymorphism (i.e., AS-3, -16, -24, -31, and -32). Chromosome identity was ascribed to unlinked markers and marker linkage groups by comparison with the respective marker affinities on existing Atlantic salmon mapping panels [45].

### QTL analysis

Single-parent analysis yielded no genome-wide significant QTL and a total of ten linkage groups were associated with chromosome-wide significant QTL across both traits (see Table 1). Na^+^/K^+^-ATPase activity QTL were found on AS-4p, -4q, -5, -14q, -19q, -22, -and -23, while blood plasma osmolality QTL were found on AS-2, -4q, -9, -12p, -12q, -14q, -19q, and -17q. Co-localization of QTL among Na^+^/K^+^-ATPase activity and blood plasma osmolality was evident on AS-4q, -14q, and -19q. Combined family analysis yielded QTL on AS-4q significant at the genome-wide level for Na^+^/K^+^-ATPase activity and the chromosome-wide level for blood plasma osmolality. The percentage of experimental variation explained by QTL for Na^+^/K^+^-ATPase activity ranged from 10.9 to 21.4%, with the greatest percentages associated with AS-14q and -22, at 20.3% and 21.4%, respectively. Variation explained by blood plasma osmolality QTL ranged from 9.4 to 30.6%, with the most explained by loci on AS-12q, -14q, and -4q, at 30.6%, 28.0%, and 21.5%, respectively.

**Table 1:** **Location of salinity tolerance QTL detected in two Atlantic salmon (*Salmo salar*) full-sib families**

**Analysis**	**Trait**	**LG**	**Parent/family**	**Marker or interval**	***P***	**PEV**
Independent parents	NKA	4q	Female/7	Ssa17DU - Ssa171DU	0.019	0.136
		4p	Female/7	OMM1105	0.033	0.120
		4q	Female/9	OMM1161 - Ssa171DU	0.027	0.169
		5	Female/9	Str58CNRS - AAG/CAC94	0.033	0.149
		14	Female/9	BHMS111 - OMM1032	0.004	0.203
		14	Male/9	BHMS111 - OMM1032	0.036	0.124
		19	Male/9	BX319411	0.046	0.109
		22	Male/7	AAG/CTT93 - AAG/CTA138	0.013	0.214
		23	Male/7	Ssa20.19NUIG - AGC/CAA121	0.04	0.151
	OSMO	2	Male/7	AAC/CAC86 - ACT/CAG243	0.031	0.149
		4q	Female/9	OMM1161 - Ssa171DU	0.01	0.215
		9	Female/7	Ssa408UoS	0.008	0.157
		12q	Female/7	OMM1108	0.031	0.122
		12p	Female/7	BHMS272	0.004	0.281
		12q	Male/7	OMM1016 - OMM1108	0.028	0.306
		14	Female/9	BHMS111 - OMM1032	0.004	0.094
		14	Male/9	BHMS111 - OMM1032	0.008	0.280
		17q	Female/7	One114ADFG - OtsG83bUCD	0.044	0.127
		19	Male/9	BX319411	0.009	0.153
Combined families	NKA	4q	Female/7	Ssa171DU	0.031^a^	0.063
			Male /7			0.131
			Female/9			0.039
			Male/9			0.121
	OSMO	4q	Female/7	Ssa171DU	0.051	0.046
			Male/7			0.224
			Female/9			0.021
			Male/9			0.022

Na^+^/K^+^-ATPase activity (NKA); blood plasma osmolality (OSMO); linkage group (LG); proportion of experimental variation (PEV). Chromosome-wide QTL (no superscipt) are significant at *P* ≤ 0.05, while genome-wide QTL (a) are significant at FDR ≤ 0.05.

### Comparative genomics with 3R species

Among the linkage groups associated with salinity tolerance QTL in Atlantic salmon we found 122, 128, 122, and 105 genetic markers exhibiting significant homology with the zebrafish (*Danio rerio*), three-spined stickleback (*Gasterosteus aculeatus*), medaka (*Oryzias latipes*), and green-spotted pufferfish (*Tetraodon nigroviridis*) genomes, respectively. Similar patterns were evident in comparisons with Arctic charr, where 90, 108, 85, and 77 genetic markers showed homology with the respective aforementioned 3R genomes. Because preliminary comparisons revealed that stickleback shared the greatest synteny with salmonid genomes, full synteny maps for Atlantic salmon, Arctic charr, and rainbow trout were constructed based on comparisons with the stickleback genome.

**Figure 1:**
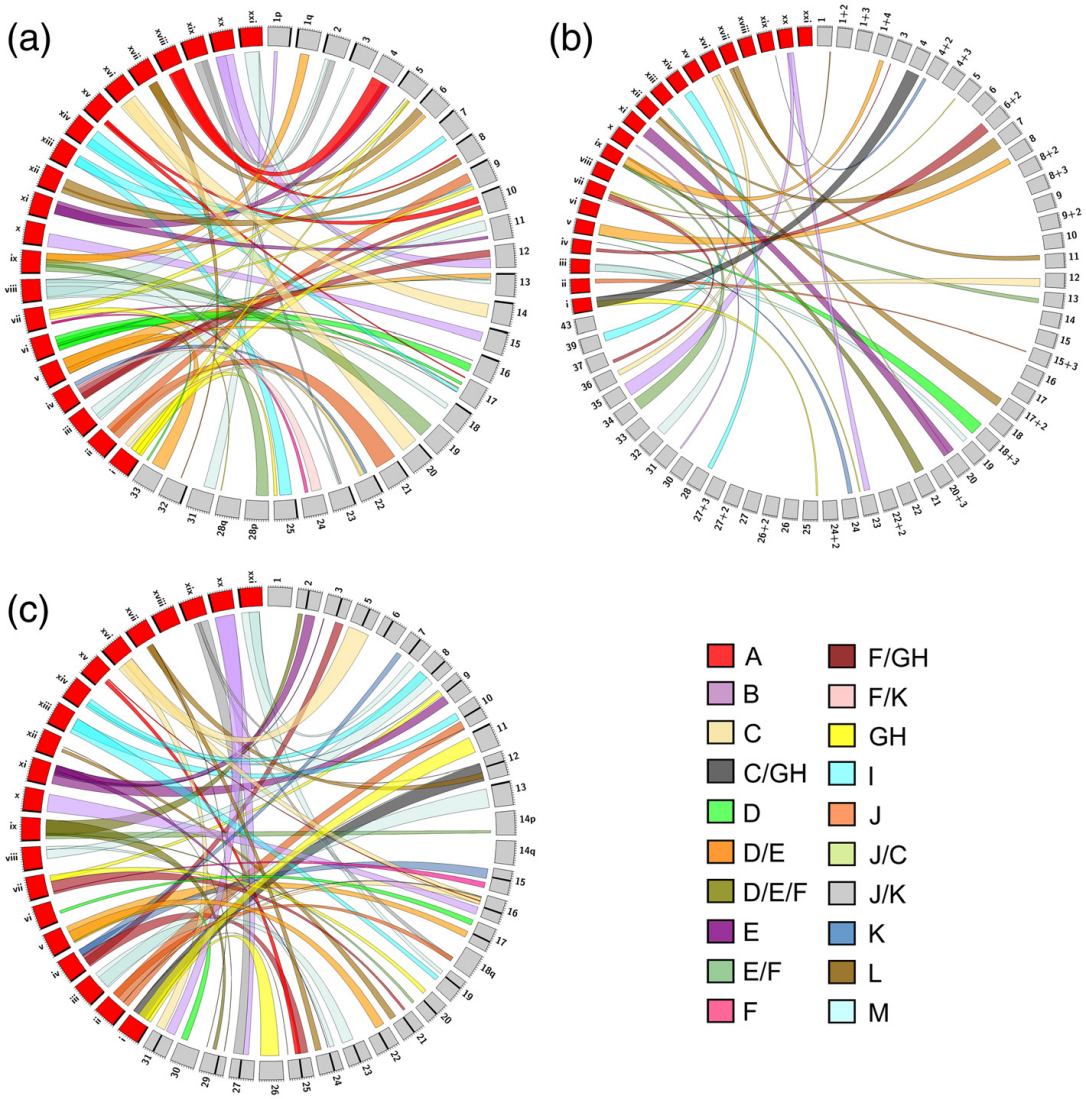
**Ideograms depicting genome synteny of (a) Atlantic salmon (*Salmo salar*), (b) Arctic charr (*Salvelinus alpinus*), and (c) rainbow trout (*Oncorhynchus mykiss*) with three-spined stickleback (*Gasterosteus aculeatus*).** Syntenic affinities with the 2R ancestral genome reconstructed by Kasahara et al. (2007) [50] are colour-coded as per the legend on the bottom right-hand corner. Synteny maps are based on the most recent published linkage maps containing information for EST accessions within each salmonid [31,45] and the BROAD S1 stickleback genome sequence from ENSEMBL v59 - v61 [60]. Salmonid linkage groups (grey) and stickleback chromosomes (red) are standardized to 100 units. Chromosomes and linkage groups are arranged in a p-arm to q-arm clockwise orientation unless otherwise noted. The positions of centromeres (black bands) on metacentric linkage groups AS-4/11/12/17/19/24/28/31/33 are undefined, as is the orientation of AS-19/28p/28q/31/33, RT-1/14p/14q/18q/26/30, and all Arctic charr linkage groups. RT-18p is not included on the reference mapping panel. Satellite marker groupings that remain unlinked to a primary linkage group segment have +2, +3 or +4 appended to their predicted linkage group affiliation. Ideograms created using Circos [63].

All linkage group arms from the most recent Atlantic salmon mapping panel [45] were syntenic with at least a single stickleback chromosome (see Figure 1a and Additional file 5). Thirteen linkage group arms had syntenic affinities with two stickleback chromosomes, (i.e., AS-2qa/Ga-VIII, AS-2qb/Ga-XIX, AS-6qa/Ga-XVII, AS-6qb/Ga-IX, AS-8qa/Ga-XVIII, AS-8qb/Ga-XII, AS-16qa/Ga-VII&VI, AS-17p/Ga-XV&VI, AS-23qa/Ga-XIX&VIII, AS-25qa/Ga-XIII, AS-25qb/Ga-I, and AS-33q/Ga-V&VI), while five arms had mosaic affinities with three stickleback chromosomes (i.e., AS-5qb/Ga-VII&I, AS-9qa/Ga-II, AS-9qb/Ga-VII, AS-9q/Ga-XIV, AS-10qa/Ga-XV, AS-10qb/Ga-IV, AS-10qc/Ga-XV, AS-13qb/Ga-V&VI&XXI, AS-17qa/Ga-V, AS-17qb/Ga-XIV&VIII, AS-22qa/Ga-IV, and AS-22qb/Ga-I&II). Atlantic salmon linkage groups designated as a, b, or c reflect linkage group arms that show patterns consistent with ancestral inter-arm fusion events, where each letter represents a segment that has homologous affinities with a different rainbow trout chromosome arm [46].

Due to the relatively low marker resolution of the Arctic charr mapping panel [31], synteny with the stickleback genome was undefined for several linkage groups (i.e., AC-9, -10, -14, -16, -17, -26, -27, -30, and -43) (see Figure 1b and Additional file 6). However, known homologies between Arctic charr and rainbow trout [31] suggest that certain syntenies can be expected: AC-10/RT-10q&18q/Ga-II&XIX, AC-14/RT-24q/Ga-XVII, AC-16/RT-8q/Ga-VIII, AC-27/RT-7q/Ga-XXI, AC-27+2/RT-12p&16p/Ga-I&II&XV&XVI, AC-27+3/RT-19p/Ga-XXI, AC-43/RT-9p/Ga-VII&XIV. No linkage group arms were syntenic with more than a single stickleback chromosome, and marker coverage to infer homologies is still largely incomplete in the salmonids as synteny blocks on seven linkage groups spanned less than 10% of the total linkage group length (i.e., AC-1, -3, -5, -15+2, -22, -31, and -37). Consequently, five stickleback chromosomes showed no syntenic affinity with any Arctic charr linkage group (i.e., Ga-VI, -VIII, -XV, -XVIII, and -XXI). Nevertheless, knowledge of synteny patterns of rainbow trout with stickleback (see Figure 1c and Additional file 7) and with Arctic charr [31] (see Additional file 8) allowed us to deduce probable homologies with Arctic charr for most of the unassigned chromosomes: Ga-VI/RT-17p/AC-8+2, Ga-VIII/RT-8q&-24p/AC-13&16, Ga-XV/RT-16p/AC-4&12&27+2, Ga-XXI/RT-7q&19p/AC-11&12&14&27&27+3.

**Figure 2:**
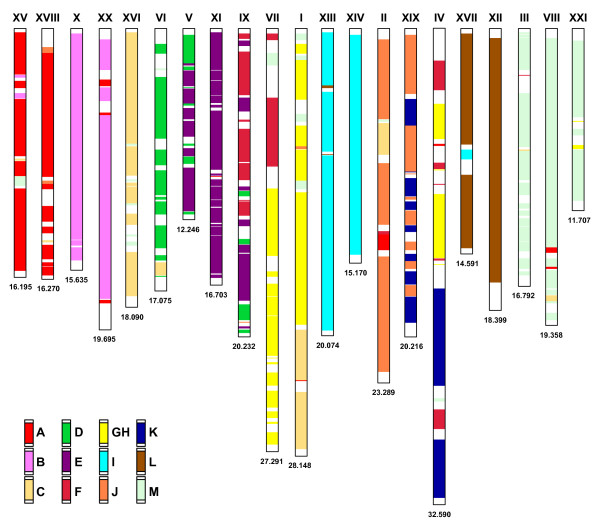
**Distribution of 2R proto-Actinopterygian ancestral chromosome affinities in the genome of three-spined stickleback (*Gasterosteus aculeatus*).** Chromosomes are all acrocentric and are arranged with centromeres at the top. 2R ancestral chromosome designations follow the convention of Kasahara et al. (2007) [50]. Syntenic affinities are colour-coded as per the legend on the bottom left-hand corner. The size of each chromosome is denoted in millions of base-pairs at the bottom of each chromosome and was obtained from ENSEMBL v60 [60].

Six linkage group arms in the rainbow trout mapping panel [45] could not be assigned a syntenic affinity with any stickleback chromosome (i.e., RT-1p/q, -6p/q, -14q, -22q), while eight linkage group arms had multiple syntenic affinities (i.e., RT-9p, -10q, -12p/q, -16p, -18, -24p, and -29q). RT-12p and -16p in particular had mosaic syntenies with three (i.e., Ga-I, -II, and XVI) and four (i.e., Ga-I, -II, -XVI, and –XV) stickleback chromosomes, respectively. Ga-XVIII was the only stickleback chromosome without syntenic affinities on any rainbow trout linkage group due to lack of marker homologies between the maps. However, Ga-XVIII is largely derived from the A ancestral lineage of teleost chromosomes (see Figure 2), and hence homologies to either RT-14q, -24q or -25q could be expected [45].

### Stickleback 3R duplicated homeologies

Several homeologous chromosome pairs have remained largely conserved in the stickleback lineage (i.e., Ga-XV/XVIII, Ga-X/XX, Ga-XIII/XIV, Ga-XII/XVII, and Ga-III/VIII/XXI) as pairs of chromosomes characterized by lineage-specific duplicated regions (see Figure 2). Several ancestral groupings are recognized as having one of the pair of chromosomes retained largely intact, with the duplicated regions within the lineage mosaically distributed along with other ancestral chromosome regions. For example, a single chromosome from the C ancestral lineage (Ga-XVI) is conserved, however the homeolgous regions from this ancestral group have been divided and arrayed on segments of Ga-I, Ga-II and Ga-VI. Similarly, chromosomes Ga-V and Ga-XIX appear to be mosaic arrangements between lineage D/E and J/K elements, respectively. Larger regions of homology within single chromosomes appear to occur with D lineage within Ga-VI and within the E lineage on Ga-XI. Similarly, Ga-II is largely derived from the J lineage, while half of Ga-IV appears to be derived from K lineage genes. Ancestral lineage GH is likely derived from a triplication event in teleosts (similar to M-lineage chromosomes) [45] and is arrayed within segments of Ga-I, -IV, and -VII, with all these chromosomes possessing larger fused segments of C, F/K, and F segments, respectively. Larger syntenic blocks from the F ancestral lineage do appear retained within Ga-IX, but even this chromosome appears to be derived from mosaic arrangements with D/E lineage genes, which may have arisen from multiple translocation and/or fusion events.

## Discussion

The detection of salinity tolerance QTL in the Atlantic salmon genome indicates that allelic variation at certain loci exerts significant effects on salinity tolerance performance traits. We found multiple chromosome-wide significant QTL for Na^+^/K^+^-ATPase activity and/or blood plasma osmolality over nine linkage groups. Among these we found a single genome-wide significant QTL on AC-4q. Co-localization of QTL for both traits occurred on AS-4q, -14q and -19q.

### Homeologous QTL patterns

The distribution of salinity tolerance QTL in Arctic charr and rainbow trout reveal several possibilities where QTL may be at homeolgous loci [11,15], however, such patterns are not predominant in Atlantic salmon. Among several linkage groups with homeologous affinities, only two QTL localized to a homeologous region that is supported by a syntenic block of duplicated loci (i.e., AS-22/23), which also contain duplicated copies of ATP1α1b [38,45]. Single marker affinities suggest that additional homeologous QTL may occur on AS-2/23 and AS-17/19 [45]. Homologies among 3R and 4R genomic segments are evident when comparisons are made with stickleback chromosomes that are syntenic with multiple Atlantic salmon linkage groups. For example, AS-2/23 share a syntenic block with Ga-XIX, as do AS-17qb/9qab with Ga-XIV, AS-5qb/9qb with Ga-VII, and AS-12p/22qa with Ga-IV.

The relative paucity of putative homeologous QTL could result from the low density of duplicated markers currently detected in the Atlantic salmon map [30,45], but may also be a function of genomic structure. Current mapping panels contain 9.5% duplicated loci for Atlantic salmon and 26% for rainbow trout, despite containing a similar contingent of type I and II markers [45]. This suggests that a smaller proportion of duplicate loci have been conserved in Atlantic salmon and may account for the paucity of homeologous QTL. Further, asymmetry in the conservation of duplicate loci may be related to the unique structure of the Atlantic salmon karyotype, which is characterized by a reduction in chromosome arms (= 72-74) that is not evident in other salmonids, including Arctic charr (= 98) and rainbow trout (= 104) [41].

### Homologous QTL patterns

Salinity tolerance in Atlantic salmon appears to be affected by some of the same genetic regions linked to QTL in other salmonids. Homologies of Atlantic salmon with Arctic charr and rainbow trout suggest that the effects of these loci may be conserved. Of the eleven linkage group arms affiliated with salinity tolerance QTL in Atlantic salmon, eight are syntenic with linkage groups containing salinity tolerance QTL in Arctic charr [15] (see Table 2), and two are syntenic with linkage groups containing similar QTL in rainbow trout [11]. QTL synteny blocks that are conserved across all three species (i.e., AS-4q/AC-20/RT-9q and AS-22/AC-12/RT-15p) provide the strongest support for the conservation of QTL effects in salmonids.

**Table 2:** **Homology among Atlantic salmon (*Salmo salar*) salinity tolerance QTL with two other salmonids**

***S. salar***	***S. alpinus***	***O. mykiss***
**LG (QTL trait)**	**LG (QTL trait)**	**LG (QTL trait)**
2 (O)	**4 (O, N, S1, S2)**, 12 (O), 27 (O), **16 (S2)**	
4q (N, O)	***20 (O, S1, S2)***	***9q (G)***
5 (N)	20 (O, S1, S2)	
9 (O)	***5 (N, S1)***, 15 (N)	
12p (O)	**8 (S2)**, 23 (S2)	25p (Cl)
14q (N,O)	20 (O, S1, S2)	
17q (O)	**28 (N)**	
19q (N,O)	***34 (S1)***	
22 (N)	1 (N, S1, S2), ***12(O)***, 27 (O), 23 (S2)	**15p (G)**
23 (N)	**12(O)**, 16 (S2), ***26 (S1, S2)***, **27 (O)**	

Salinity tolerance QTL associated with Na^+^/K^+^-ATPase activity (N), blood plasma osmolality (O), blood plasma Cl- concentration (Cl), gill tissue weight (G), and weight-specific growth rates at time S1 and S2. Homology assignments are based on > 2 markers (bold, italics), 2 markers (bold), or a single marker. Linkage group (LG) arms for Arctic charr are undefined. All QTL are chromosome-wide significant. Genome-wide significant QTL are underlined. Data for rainbow trout (*Oncorhynchus mykiss*) and Arctic charr (*Salvelinus alpinus*) QTL were obtained from Le Bras et al. (2011) [11] and Norman et al. (2011) [15], respectively.

### Distribution of candidate genes in salmonid genomes

**Figure 3:**
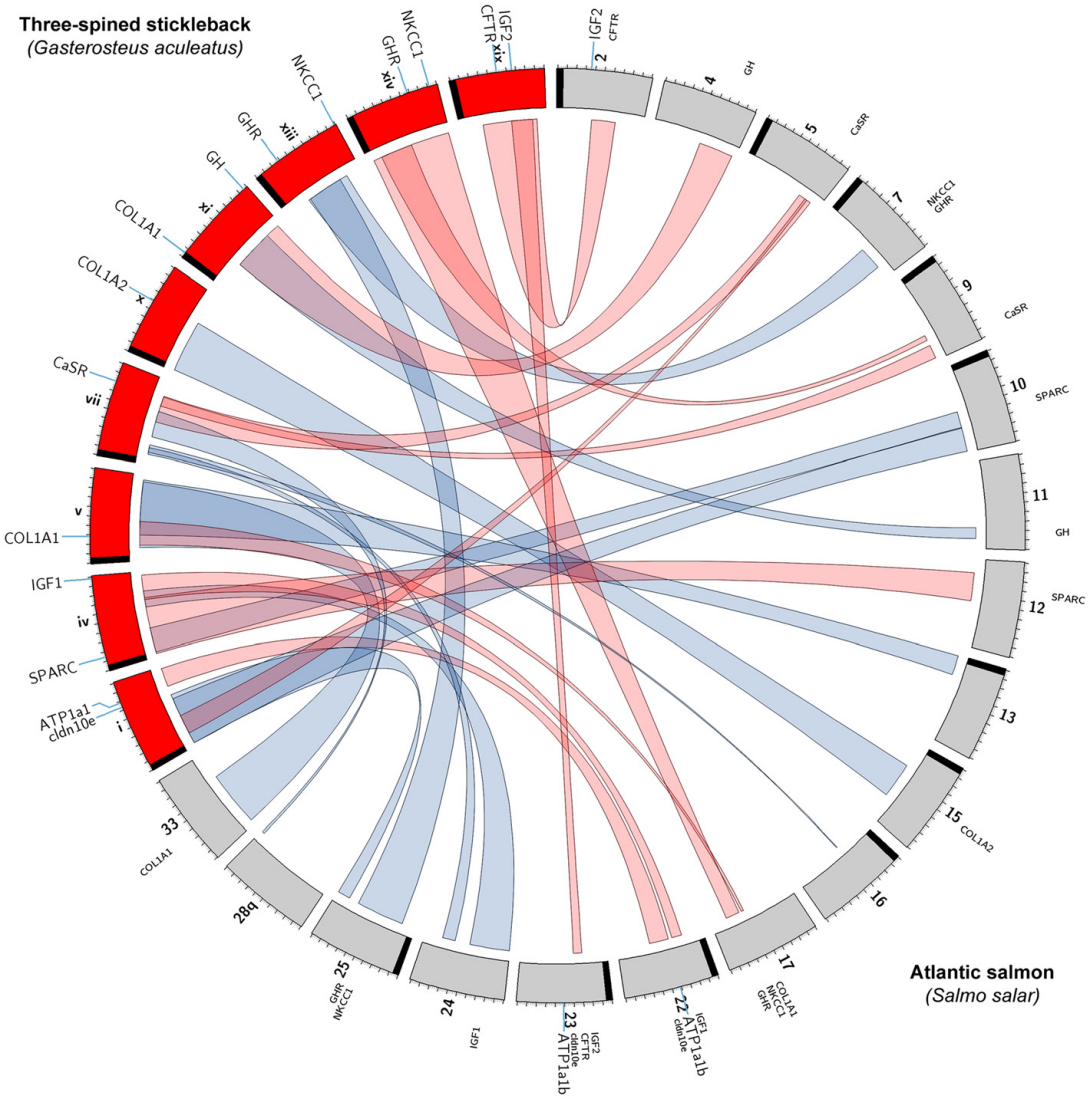
**Synteny patterns between the Atlantic salmon (*Salmo salar*) mapping panel and chromosomes that contain salinity tolerance candidate genes in three-spined stickleback (*Gasterosteus aculeatus*).** Synteny was established using the Atlantic salmon linkage map [45] and the BROAD S1 stickleback genome sequence from ENSEMBL v59 - v61 [60]. Atlantic salmon linkage groups (grey) and stickleback chromosomes (red) are standardized to 100 units. Red synteny bands indicate linkage groups that contain salinity tolerance QTL. Chromosomes and linkage groups are arranged in a p-arm to q-arm clockwise orientation unless otherwise noted. The positions of centromeres (black bands) on metacentric linkage groups AS-4/11/12/17/24/28q are undefined, as is the orientation of AS-28q/33. Candidate genes include: Na^+^/K^+^-ATPase α-isoform (ATP1α1) and -α1b isoform (ATP1α1b), calcium-sensing receptor (CaSR), cystic fibrosis transmembrane conductance regulator-like anion channel (CFTR), claudin isoform 10e (cldn10e), collagen type 1 alpha 1 (COL1A1) and alpha 2 (COL1A2), growth hormone (GH), growth hormone receptor (GHR), insulin-like growth factors 1 (IGF1) and 2 (IGF2), Na^+^/K^+^/2Cl^-^ isoform 1 (NKCC1), and secreted protein, acidic, rich in cysteine (SPARC). The precise positions of candidate genes on stickleback chromosomes are denoted by connection lines (blue), as are genes mapped in Atlantic salmon [12,38]. Predicted gene positions on Atlantic salmon linkage groups are represented by labels without connection lines. IGF1 and NKCC1 map to AS-24 [12] and AS-17q [15], respectively, however their precise positions are unknown. Ideogram created using Circos [63].

Gene location predictions derived from comparative synteny maps of stickleback with Atlantic salmon, Arctic charr, and rainbow trout reveal three instances where Atlantic salmon linkage groups associated with salinity tolerance QTL may possess candidate genes in unique combinations not evident in the other species (i.e., AS-17, -22, and -23) (see Figure 3 and Additional file 9 and Additional file 10). In the first instance, AS-23 is predicted to contain copies of cldn10e, IGF2, and CFTR in addition to ATP1α1b, which has been mapped to AS-22/23 [38]. In both Arctic charr and rainbow trout, this putative cluster is broken up into two distinct gene-pair groups: ATP1α1b/cldn10e and IGF2/CFTR. Synteny maps suggest that ATP1α1b/cldn10e and IGF2/CFTR reside on separate arms of the metacentric AC-4 in Arctic charr, while only the arm that is syntenic with ATP1α1b/cldn10e is associated with a QTL. Previous studies predict that AC-12/27 contain duplicate copies of ATP1α1b and cldn10e, however extensive synteny of AC-4 with Ga-I, where orthologues of ATP1α1b and cldn10e are located, suggests that these genes may instead reside on AC-4. Regardless, salinity tolerance QTL are associated with AC-4, -12 and -27 [15]. Duplicate copies of ATP1α1b map to RT-12q/16p in rainbow trout [37]. These locations are consistent with the predicted positions of ATP1α1b/cldn10e based on synteny with stickleback, however RT-12q/16p were not associated with salinity tolerance QTL [11]. It is unclear if this discordance can be attributed to biological differences or experimental design, given that Le Bras et al. (2011) measured different performance indicators than in the current study and those used for Arctic charr [15]. Nevertheless, the importance of these genes to salinity tolerance is well documented [4,24,26,47,48].

The second novel candidate gene cluster was located on AS-22, where IGF1 and cldn10e duplicates were predicted to occur in conjucntion with a mapped duplicate of ATP1α1b. IGF1 is located on AC-3/24 in Arctic charr [12], though neither linkage group has been reported to be associated with a salinity tolerance QTL [15]. IGF1 duplicates in rainbow trout are located on RT-7p/15p, and only RT-15p is linked with a QTL. A copy of IGF1 is located on AS-24 [12], which is homeologous to AS-22 [30]. Though experiments clearly show that IGF1 is involved in hypo-osmoregulation [49], evidence that allelic variation at IGF1 loci significantly affects salinity tolerance capacity remains equivocal. The third and final novel candidate gene cluster was located on AS-17q, where GHR and COL1A1 are predicted to co-occur with a mapped NKCC locus [15]. The linkage between NKCC1 and GHR is likely conserved among AS-17qb, AC-28 in Arctic charr, and RT-10p, RT-19q in rainbow trout, all of which are associated with salinity tolerance QTL. However, a segment on AS-17qa is also syntenic with a region on Ga-V that contains COL1A1. The segment of AS-17qb bearing NKCC1 and GHR derives from the I teleost chromosome lineage, while the segment predicted to possess COL1A1 on AS-17qa would appear to derive from the D lineage [45]. COL1A1 duplicates are located on chromosomes Ga-V and Ga-XI in stickleback suggesting evolutionary origins to the E ancestral lineage of fishes. However, since both D and E chromosome blocks appear to show mosaic affinities with one another in model teleost species including salmonids [45,50] it is possible that similar regions exist on AS-17qa that are currently undetected.

The positions of several other salinity tolerance QTL in Atlantic salmon are consistent with the predicted locations of candidate genes. Synteny of AS-5/9 with a segment containing CaSR on Ga-VII suggests that duplicate copies of CaSR could underlie QTL on those linkage groups. Multiple CaSR transcripts have been detected in Atlantic salmon, however it remains unclear if they are transcribed from separate loci or represent a collection of alternate splicing variants transcribed from a single locus. Similar patterns are evident in Arctic charr, however, presently only AC-5 is syntenic with CaSR on Ga-VII. This might be due to the low marker resolution of the Arctic charr mapping panel or may suggest that only a single CaSR locus is conserved in Arctic charr. In rainbow trout duplicate copies of CaSR appear to reside on RT-9p/20p. Though neither of these linkage group arms is associated with a salinity tolerance QTL, a major smoltification QTL has been linked to RT-20q in rainbow trout [51], which shares a syntenic block with AS-19q. This is particularly interesting as CaSR is an osmosensor that is upregulated during smoltification in Atlantic salmon [20].

Synteny with other salmonids and stickleback strongly suggests that a copy of GH is located on AS-4q, where we detected the only genome-wide significant QTL for salinity tolerance. Further, salinity tolerance QTL map to AC-20 [15] and RT-9q [11], where GH has been mapped in Arctic charr [14] and rainbow trout [13], respectively. These findings clearly suggest that these effects, which can tentatively be attributed to genetic variation at GH loci, are conserved in salmonids from three different genera. Lastly, synteny with Ga-IV suggests that a QTL on AS-12p could be related to a SPARC locus. This is corroborated by a QTL on RT-25p, which is also syntenic with the position of SPARC on Ga-IV. There is currently no synteny block in Arctic charr that overlaps with SPARC on Ga-IV. However, we have tentatively assigned SPARC to AC-6+2, as it is syntenic with a region that is proximal to SPARC on Ga-IV, although this region does not contain a reported salinity tolerance QTL [15].

### Candidate gene evolution in 3R and 4R genomes

The relative arrangements of candidate genes in the 4R derivative salmonids have for the most part remained unchanged from patterns in the reconstructed proto-Actinopterygian 2R genome (see Table 3). Unfortunately, the low resolution of genetic maps for salmonids has precluded a precise designation of synteny block boundaries on stickleback chromosomes. Where such boundaries fell between candidate gene positions on 3R chromosomes we could not confidently designate the pertinent genes as unlinked in the 4R genome in question (e.g., IGF2 and CFTR on Ga-XIX are intersected by a synteny block on AS-23; see Figure 3). Therefore our predictions likely underestimate the extent that genomic rearrangements have altered the relative positions of candidate genes. Nevertheless, our findings suggest several cases where candidate genes were paired in the 2R ancestor and remained so in both 3R and 4R genomes (e.g., IGF2/CFTR, NKCC1/GHR, GH/COL1A1, and ATP1α1b/cldn10e).

**Table 3:** Summary of predicted locations of salinity tolerance candidate genes on linkage groups from three salmonids

**Gene Name**	**2R**	**3R**	**4R**	***S. alpinus***	***O. mykiss***
		***G. aculeatus***	***S. salar***		
ATP1α1b	C	I	22^a^/23^ab^	4/12	12q^a^/16p^a^
CaSR	GH	VII	5/9	5	9p/20p
CFTRI & II	J/K	XIX	2/23	4^d^/19^d^	10q/18q/27q
cldn10e	C	I	22/23^c^	4/12	12q16q
COL1A1	E	XI	?	20	9q
	E	V	17q/33	8	17q/22p
COL1A2	B	X	15	31	16q
GH1 & 2	E	XI	4q/11q	20^a^	2q/9q^a^/24
GHR	I	XIII	7/25	39	10p/19q
	I	XIV	17q	28	8p
IGF1	K	IV	22/24^a^	3^a^/24^a^	7p^a^/15p^a^
IGF2	J/K	XIX	2^a^/23	4^a^/19^a^	10q/18q/27q^a^
NKCC1	I	XIII	7/25	?	10p/19q
	I	XIV	17q^a^	28	?
SPARC	F/G	IV	10/12p	6	3q/25p

Salinity tolerance candidate genes included in comparisons were: Na^+^/K^+^-ATPase α1b isoform (*ATP1α1b*), calcium-sensing receptor (*CaSR*), cystic fibrosis transmembrane conductance regulator-like anion channel isoforms I and II (*CFTRI/II*), claudin isoform 10e (cldn10e), collagen type 1 alpha 1 (*COL1A1*) and alpha 2 (*COL1A2*), growth hormone 1 and 2 (*GH1 & 2*), growth hormone receptor (*GHR*), insulin-like growth factors 1 (IGF1) and 2 (IGF2), Na^+^/K^+^/2Cl^-^ isoform 1 (*NKCC1*), and secreted protein, acidic, rich in cysteine (*SPARC*). Synteny blocks were defined from comparative genomics of Atlantic salmon (*Salmo salar*), Arctic charr (*Salvelinus alpinus*), and rainbow trout (*Oncorhynchus mykiss*) with the stickleback (*Gasterosteus aculeatus*) genome. Some genes have already been mapped in 4R genomes (a): ATP1α1b [37,38]; GH [13,14]; IGF1 and IGF2 [12]; NKCC1 [15]. Mapped genes are included where synteny between 3R and 4R genomes is not apparent (b). In certain cases gene homology was assigned based on close proximity with ATP1α1b (c) or IGF2 (d) in the stickleback genome.

AS-17pq, -22, and −23 have homologous affinities with several regions of the stickleback genome which is consistent with findings that show these linkage groups are a product of multiple fusion and translocation events [46]. Accordingly, certain candidate genes that are unlinked in 2R and 3R genomes appear to be linked in unique combinations in Atlantic salmon. For instance a cluster on AS-17q (i.e., COL1A1/NKCC1/GHR) contains COL1A1, which is homologous with Ga-V and derived from the 2R ancestral E grouping (i.e., 2R-E). Similarly, NKCC1/GHR are homologous with 2R-I and Ga-XIV. In addition, part of a conserved cluster on AS-22 (i.e., ATP1α1b/cldn10e) is derived from 2R-C and homologous with Ga-I, while the other part of the cluster (i.e., IGF1) is derived from 2R-K and homologous with Ga-IV. Finally, IGF/CFTR duplicates may be linked with ATP1α1b/cldn10 on AS-23, which has homeologous affinities with AS-22. IGF2/CFTR are derived from 2R-J/K and homologous with Ga-XIX. Although large tracts of AS-23 are currently unassigned to ancestral 2R origins, AS-22 appears largely derived from 2R-C/K origins.

Comparative synteny maps of Atlantic salmon, Arctic charr, and rainbow trout with the stickleback genome reveal that disparity in salinity tolerance capacity in salmonids is correlated with variation in genomic structure. Though we found no difference in putative gene cluster patterns between Arctic charr and rainbow trout, several factors lead to the hypothesis that unique gene clusters predicted for AS-17q, -22, and -23 could be related to the superior salinity tolerance capacity in Atlantic salmon. This finding is consistent with observations that genes are not randomly arranged in eukaryotic genomes [42], but are instead arranged in clusters that tend to be coexpressed [43,52] and co-methylated [53]. A logical prediction from such patterns is that the products of clustered genes would be involved in the same biochemical pathway [44]. In terms of our results, this suggests that the benefit of unique candidate gene clusters on AS-17q, -22, and −23 could be that they are coexpressed, which posits a possible mechanism to explain patterns of increased anadromy in salmonids with reduced chromosome numbers [41]. Our hypothesis that the reduction in chromosome arm numbers in Atlantic salmon could have consequences for salinity tolerance is consistent with patterns showing that this species possesses unique clusters of candidate genes. However, until it can be tested by gene mapping efforts and expression studies, this hypothesis will remain tentative.

## Conclusion

We identified salinity tolerance QTL on multiple linkage groups in Atlantic salmon. Evidence from comparisons with Arctic charr and rainbow trout indicates that some QTL may be conserved across species, suggesting that salinity tolerance in different salmonid species is to some extent controlled by the same loci. Many QTL in Atlantic salmon were on linkage groups that shared syntenic affinities with candidate gene locations in the stickleback genome. Synteny maps revealed that candidate genes may often occur in pairs on chromosomes, patterns that were generally conserved in 2R, 3R and 4R genomes. However, we found evidence that three Atlantic salmon linkage groups may contain larger syntenic combinations of candidate genes that are not evident in any of the other 2R, 3R or 4R genomes we examined. These putative novel candidate gene clusters could have resulted from ancestral chromosome arm tandem fusion events that resulted in the current Atlantic salmon karyotype. Finally, we hypothesized that the superior hypo-osmoregulatory efficiency that is characteristic of Atlantic salmon could be related to these clusters. This hypothesis can be tested by gene mapping efforts and expression studies.

## Methods

### Strain background and rearing

Twenty-two full-sib families from the St. John River strain were produced on November 22, 2000, at the Atlantic Salmon Broodstock Development Program facilities (Chamcook, New Brunswick, Canada). Details of the rearing history of this strain are outlined in Quinton et al. (2005) [54]. At approximately ten months post-fertilization a preliminary seawater tolerance experiment was conducted on 20 individuals from each family to establish family-specific salinity tolerance capacity. Fish were rapidly transferred to seawater and mortality was monitored over a three week period. A subset of eleven families (herein numbered 1 to 11) collectively representing the full spectrum of mortality (i.e., 0 to 85%) was selected for further experimentation. In November 2001, all fish from 11 families were marked by heat branding to family. Prior to all sampling, fish were anaesthetized with tricaine methanesulfonate (MS 222; 150 mg·L^–1^). Approximately 1 month prior to the salinity tolerance experiment each tank was fitted with 100 W incandescent bulbs and illuminated 24-hours day^-1^ to initiate early smoltification in freshwater [55].

### Experimental protocol

On December 15, 2001 all fish were transported to the Huntsman Marine Science Centre (St. Andrews, New Brunswick, Canada) and transferred directly to a single aerated 3500 L fiberglass tank holding 100% seawater (36%, flow rate 26 L·min^-1^, dissolved O_2_ saturation ≥ 95%). Prior to transfer, fish were fasted for 24 hours. Ninety-six hours post-full SW exposure, blood was collected by caudal puncture with a pre-heparinized syringe (500 U mL^-1^ heparin) fitted with a 16-guage needle, aspirated into a 1.5 mL centrifuge tube, and placed on ice. After each blood sample was collected, each fish was euthanized by cranial impact and its second and third gill arches quickly excised to a 1.5 mL cryovial and frozen in liquid nitrogen at for future analysis. When 20 fish had been sampled, blood was centrifuged at 5000 g for eight minutes at 4°C and plasma transferred to a new 1.2 mL cryovial and preserved in liquid nitrogen. Caudal fin (progeny) or kidney (parents) tissue was collected for genetic analysis. All samples were stored at -80°C.

### Phenotypic measurements

Blood plasma osmolality (mOsmol·kg^-1^) was measured using a vapour pressure osmometer (Wescor model 5520; Wescor Inc., Utah, USA). Na^+^/K^+^-ATPase activity (μmol ADP·mg protein^-1^·h^-1^) was determined spectrophotometrically following the modified methods of McCormick (1993) [56]. Gill filaments were homogenized on ice in SEI buffer (150 mM sucrose, 10 mM EDTA, 50 mM imidazole) with a ground glass homogenizer. Homogenates were centrifuged at 5000 g for 30 s at 4°C to separate insoluble material from the supernatant, which was used directly in the assay mixture (100 mM NaCl, 20 mM KCl, 5 mM MgCl_2_, 50 mM imidazole, 3 mM ATP, 2 mM phospho(enol)pyruvate, 0.2 mM NADH, with pyruvate kinase and lactic dehydrogenase added to excess) or the assay mixture plus ouabain (10 mM), a Na^+^/K^+^-ATPase enzyme inhibitor. Na^+^/K^+^-ATPase activity was measured in duplicate at 340 nm for 10 minutes using a temperature controlled Cary 300 microplate reader (Agilent Technologies) maintained at 10°C. Enzyme activity was corrected for assay time and total sample protein. Protein concentration was determined with a BioRad protein assay kit (BioRad Laboratories, Inc.).

### Genetic marker analysis and linkage map construction

Families 7 and 9 were selected for genome mapping and QTL analysis for they exhibited particularly large variances in blood plasma osmolality and Na^+^/K^+^-ATPase activity. DNA was extracted using a standard phenol-chloroform protocol [57]. Polymerase chain reaction (PCR) amplification, electrophoresis, and DNA fragment visualization was performed following the methods of Woram et al. (2003) [58]. AFLP analysis was conducted as described by Vos et al. (1995) [59], and loci were named following the convention of Woram et al. 2004 [14].

Due to the large differences in recombination rates between males and females [13,14,30], sex-specific linkage maps were constructed using 83 microsatellite markers, 89 AFLP loci, and 3 type-I gene markers, though not all loci were informative for each mapping panel. Genetically linked markers and their relative order within linkage groups were established using several modules within the LINKMFEX software package (v2.3) (LINKFMEX, LINKGRP, MAPORD, MAPDIS) [60]. Linkage was assigned based on minimum logarithm of odds (LOD) scores of 3.0 and 4.0 for males and females, respectively.

### QTL analysis

Traits were tested for confirmation to normality (Kolmogorov-Smirnov and Lilliefors tests) and normalized as necessary with standard transformations. To remove the effect of sampling order on blood plasma osmolality, residual values (ANOVA) were used in the QTL analysis (SPSS, IBM Corporation, 2010).

MultiQTL (v2.5) software ( http://www.multiqtl.com) was used to conduct marker interval analyses, which were performed independently for each trait and parent. In a more conservative test, data for all parents from both families were combined and assessed with single marker analysis for each trait independently. Interval distances among parental maps were highly variable due to large sex-specific differences in recombination rates. This was also due to random marker positions missing within the different parents used due to chance homozygous genotypes present in these out bred parents. Therefore, it was not possible to perform the combined family analysis using interval analysis. Chromosome-wide LOD thresholds were determined empirically with 1000 permutations of the trait data against the genotypes [61]. QTL deemed significant at a chromosome-wide threshold of P ≤ 0.05 were further assessed for genome-wide significance using a B-H False Discovery Rate (FDR) test (α = 0.05).

### Comparative genomics

Using recent genetic maps published for Atlantic salmon, rainbow trout [45], and Arctic charr [31], we used the BlockON, BlockONmerge, and markerSORT components of the LINKMFEX software package (v2.3) [60] to generate updated synteny maps among these species, which allowed us to establish if any salinity tolerance QTL were located in homologous regions. Comparative analysis with the sequenced genomes of 3R model teleosts was performed as described by Danzmann et al. [45] with some modifications. We used the Distant Homologies BLASTN algorithm in ENSEMBL v59 - v61 [62] to assess the homology of genetic markers (LATESTGP DNA database) on linkage groups associated with QTL for salinity tolerance traits in Atlantic salmon, Arctic charr [15], and rainbow trout [11] with zebrafish (Zv8 and Zv9), three-spined stickleback (BROAD S1), medaka (Medaka 1.0), and green-spotted pufferfish (TETRAODON 8.0). Alignments yielding expectation values ≤ 10^-4^ and identity values ≥ 60% were accepted as homologous. To determine if linkage groups associated with salinity tolerance QTL shared homology with regions containing candidate genes in the 3R stickleback genome we aligned complete or partial mRNA sequences from Atlantic salmon for CaSR-1, -2 and -3, CFTR-I and -II, cldn10e, COL1A1 and COL1A2, GH1 and GH2, IGF-II, NKCC1a, and SPARC. Sequences for ATP1α1b [63] were obtained from zebrafish, and GHR1 and GHR2 [64] from rainbow trout (see Table 4). Ideograms were generated using Circos [65].

**Table 4:** **Genomic positions of salinity tolerance candidate genes in stickleback (*Gasterosteus aculeatus*)**

**Species**	**Locus**	**GenBank Accession**	**Source**	**GA Chr.**	**Mbp start**	**EV**	**%ID**	**ENSEMBL Annotation**	**ENSEMBL Gene No.**
*D. rerio*	ATP1α1b	AY008375	Rajarao et al. 2001	i	21.699	0	78	atp1α1	ENSGACG00000014324
*S. salar*	CaSR-1	AY245445	unpublished	vii	22.314	0	81	CaSR	ENSGACG00000020687
	CaSR-2	AY245443	unpublished	vii	22.314	0	81	CaSR	ENSGACG00000020687
	CaSR-3	AY245444	unpublished	vii	22.314	0	81	CaSR	ENSGACG00000020687
*S. salar*	CFTRI	AF155237	Chen et al. 2001	xix	10.185	0	83	CFTR	ENSGACG00000009039
	CFTRII	AF161070	Chen et al. 2001	xix	10.185	0	83	CFTR	ENSGACG00000009039
*S. salar*	cldn10e	BK006391	Tipsmark et al. 2008	i	20.691	-87	78	novel	ENSGACG00000013985
*S. salar*	COL1A1	CK873772	Seear et al. 2010	v	3.438	-95	86	COL1A1	ENSGACG00000003361
				xi	0.910	-78	87	COL1A1	ENSGACG00000005143
*S. salar*	COL1A2	EG649361	Seear et al. 2010	x	9.261	-59	88	COL1A2	ENSGACG00000006511
*S. salar*	GH1	EU621898	Von Shalburg et al. 2008	xi	16.069	-21	66	novel	ENSGACG00000014829
	GH2	EU621899	Von Shalburg et al. 2008	xi	16.069	-19	66	novel	ENSGACG00000014829
*O. mykiss*	GHR1	AY861675	Very et al. 2005	xiv	10.388	-161	69	GHR	ENSGACG00000017924
	GHR2	AY751531	Very et al. 2005	xiv	10.388	-163	72	GHR	ENSGACG00000017924
*S. salar*	IGF2	EF432854	unpublished	xix	13.287	-121	83	IGF2	ENSGACG00000011125
*S. salar*	NKCC1a	DQ864492	Mackie et al. 2007	xiv	13.969	0	82	NKCC1	ENSGACG00000018343
				xiii	19.857	0	76	NKCC1	ENSGACG00000014721
*S. salar*	SPARC	BT045906	Leong et al. 2010	iv	4.486	-171	83	SPARC	ENSGACG00000016847

Sequences from zebrafish (*Danio rerio*), Atlantic salmon (*Salmo salar*) and rainbow trout (*Oncorhynchus mykiss*) were aligned in ENSEMBL (v59 - v61) [60] (BLASTN algorithm; CDNA_ALL database; Distant homologies search sensitivity), against the genome of three-spined stickleback (GA) (BROADS1). Mbp (= bp x 10^-6^); *EV* = E-value exponent; %ID = percent similarity.

To identify homeologous stickleback chromosomes, the stickleback genome database including only protein-coding loci was obtained from ENSEMBL v60 [62]. Paralogous loci were sorted and any paralogues that exhibited < 50% homology were removed from the dataset. Using a Visual Basic program written explicitly for this task, paralogous loci were ranked from highest to lowest homology against each source chromosome gene position. A tolerance of 5% was applied and only matches that were within 5% of the top ranked match were retained. Only blocks consisting of two or more contiguous paralogous loci were considered (i.e., synteny blocks), while all single hit paralogous loci were ignored (see Additional file 11).

Construction of a synteny map of stickleback with the reconstructed proto-Actinopterygian 2R-ancestral genome began by the identification of syntenic regions between stickleback and medaka genomes. The medaka genome was used as a proxy for the 2R ancestral genome because it contains no major rearrangements since its divergence from a common ancestor with the 2R proto-Actinopterygian ancestor [50]. Genome databases for both species were obtained from ENSEMBL v60 [62]. The BlockON module from the LINKMFEX software package (v2.3) [60] was used to identify homologous loci. Only blocks of two or more contiguous orthologous loci were considered, while all single hit orthologues were ignored.

## Competing interests

No competing interests are declared by any of the contributing authors.

## Authors’ contributions

This study was conceptualized by RGD, MMF, and JDN. MR conducted the salinity tolerance trials, performed the genome scans and created the linkage maps. JDN conducted the QTL analysis, while JDN and RGD performed the bioinformatics analyses. JDN wrote the manuscript with advice from MMF and RGD. BG oversaw the rearing and maintenance of fish. All authors read and commented on the manuscript.

## Supplementary Material

Additional file 1 Genetic linkage map for family 7 female. Click here for file

Additional file 2 Genetic linkage map for family 7 male. Click here for file

Additional file 3 Genetic linkage map for family 9 female. Click here for file

Additional file 4 Genetic linkage map for family 9 male. Click here for file

Additional file 5 **Marker homology among Atlantic salmon (*Salmo salar*) and three-spined stickleback (*Gasterosteus aculeatus*).** Click here for file

Additional file 6 **Marker homology among Arctic charr (*Salvelinus alpinus*) and three-spined stickleback (*Gasterosteus aculeatus*).** Click here for file

Additional file 7 **Marker homology among rainbow trout (*Oncorhynchus mykiss*) and three-spined stickleback (*Gasterosteus aculeatus*).** Click here for file

Additional file 8 **Marker homologies among Atlantic salmon (*Salmo salar*), Arctic charr (*Salvelinus alpinus*), and rainbow trout (*Oncorhynchus mykiss*).** Click here for file

Additional file 9 **Candidate gene predictions in Arctic charr (*Salvelinus alpinus*) based on synteny with three-spined stickleback (*Gasterosteus aculeatus*).** Click here for file

Additional file 10 **Candidate gene predictions in rainbow trout (*Oncorhynchus mykiss*) based on synteny with three-spined stickleback (*Gasterosteus aculeatus*).** Click here for file

Additional file 11 **Paralogous genes in three-spined stickleback (*Gasterosteus aculeatus*).** Click here for file
