# Supplementary material for: Genomic arrangement of salinity tolerance QTLs in salmonids: A comparative analysis of Atlantic salmon (Salmo salar) with Arctic charr (Salvelinus alpinus) and rainbow trout (Oncorhynchus mykiss)
Source: BMC Genomics. 2012 Aug 24;13:420. doi: 10.1186/1471-2164-13-420 (PMC3480877; doi:10.1186/1471-2164-13-420)
Supplement: Additional file 1 — Genetic linkage map for family 7 female. [file 1471-2164-13-420-S1.pdf]

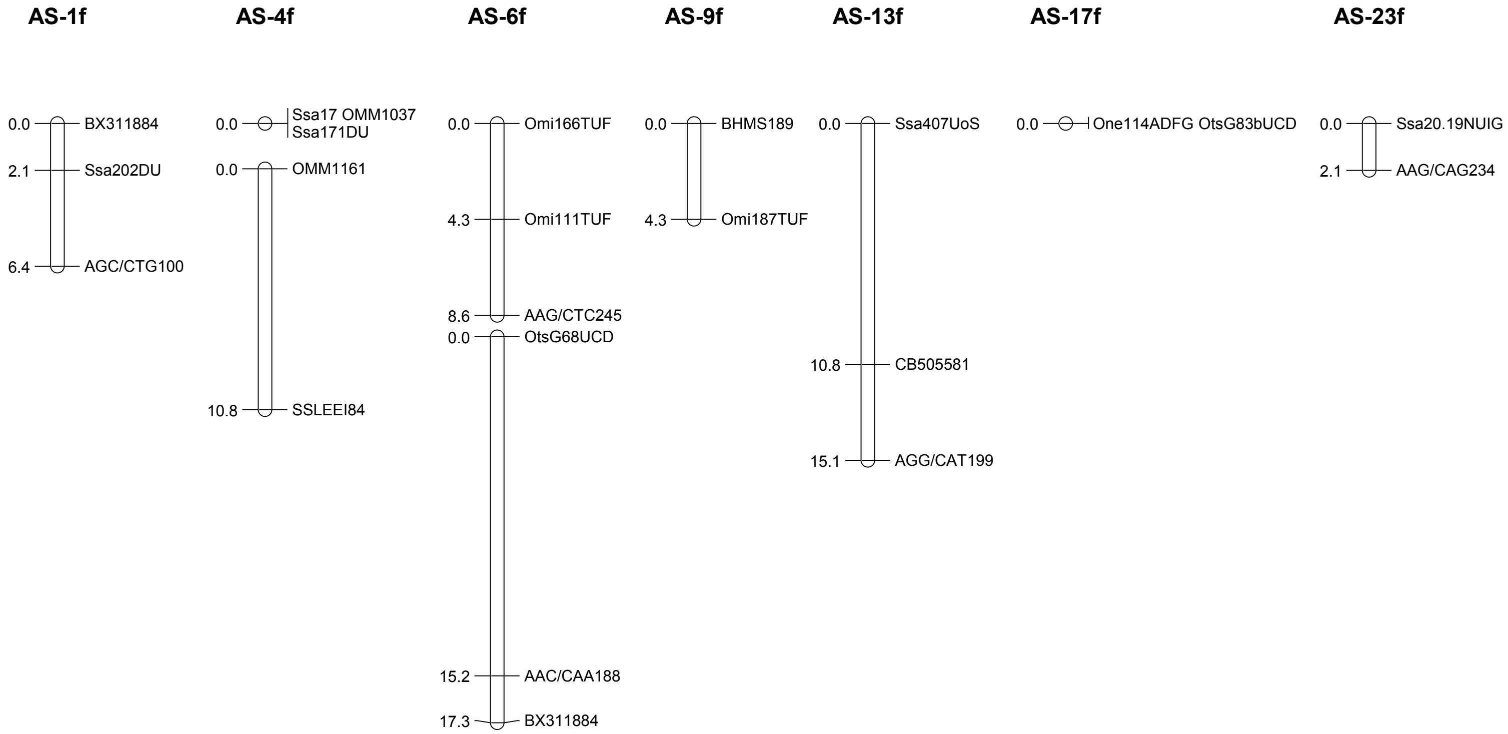

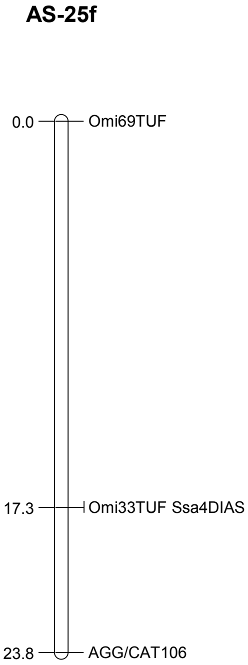

Linkage group designations for unlinked markers

Linkage Group	Marker
AS-2f	BHMS417
AS-4f	OMM1105
AS-5f	BHMS7.017
AS-6f	BHMS146
AS-7f	BHMS103
AS-7f	BHMS117
AS-8f	Ssa197DU
AS-9f	Ssa408UoS
AS-9f	BHMS221
AS-9f	Ssa413UoS
AS-9/21f	BX867151
AS-11f	OmyRGT32TUF
AS-12f	OMM1108
AS-12f	BHMS272
AS-12f	OMM1189
AS-14f	OMM1032
AS-14f	OtsG249UCD
AS-15f	AJ425048
AS-33f	CA368462
