# Supplementary material for: Genomic arrangement of salinity tolerance QTLs in salmonids: A comparative analysis of Atlantic salmon (Salmo salar) with Arctic charr (Salvelinus alpinus) and rainbow trout (Oncorhynchus mykiss)
Source: BMC Genomics. 2012 Aug 24;13:420. doi: 10.1186/1471-2164-13-420 (PMC3480877; doi:10.1186/1471-2164-13-420)
Supplement: Additional file 2 — Genetic linkage map for family 7 male. [file 1471-2164-13-420-S2.pdf]

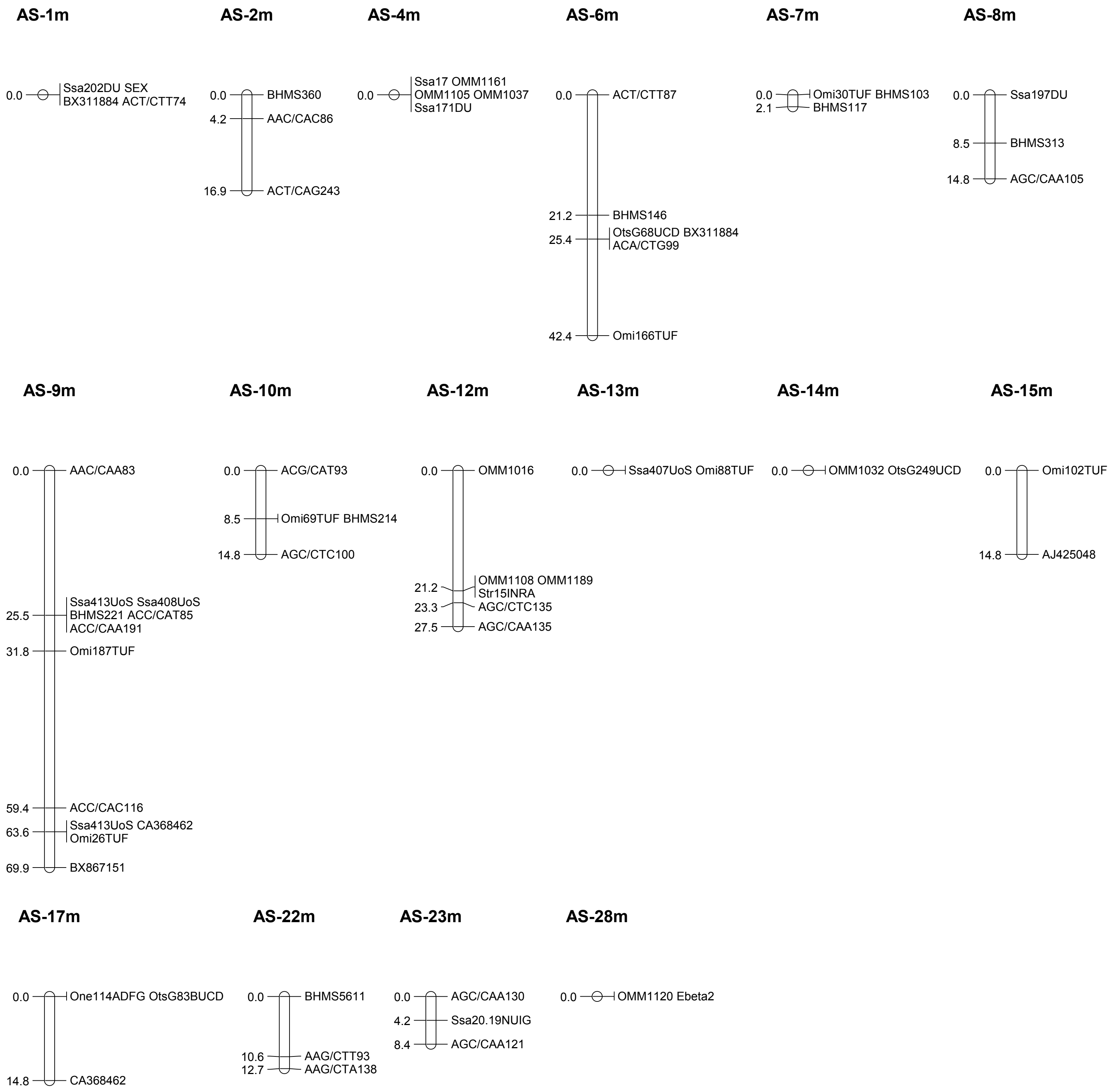

Linkage group designations for unlinked markers

Linkage Group	Marker
AS-2m	Omi85TUF
AS-5m	Ssa402UoS
AS-5m	BHMS7.017
AS-18m	BHMS420
