# Supplementary material for: Genomic arrangement of salinity tolerance QTLs in salmonids: A comparative analysis of Atlantic salmon (Salmo salar) with Arctic charr (Salvelinus alpinus) and rainbow trout (Oncorhynchus mykiss)
Source: BMC Genomics. 2012 Aug 24;13:420. doi: 10.1186/1471-2164-13-420 (PMC3480877; doi:10.1186/1471-2164-13-420)
Supplement: Additional file 3 — Genetic linkage map for family 9 female. [file 1471-2164-13-420-S3.pdf]

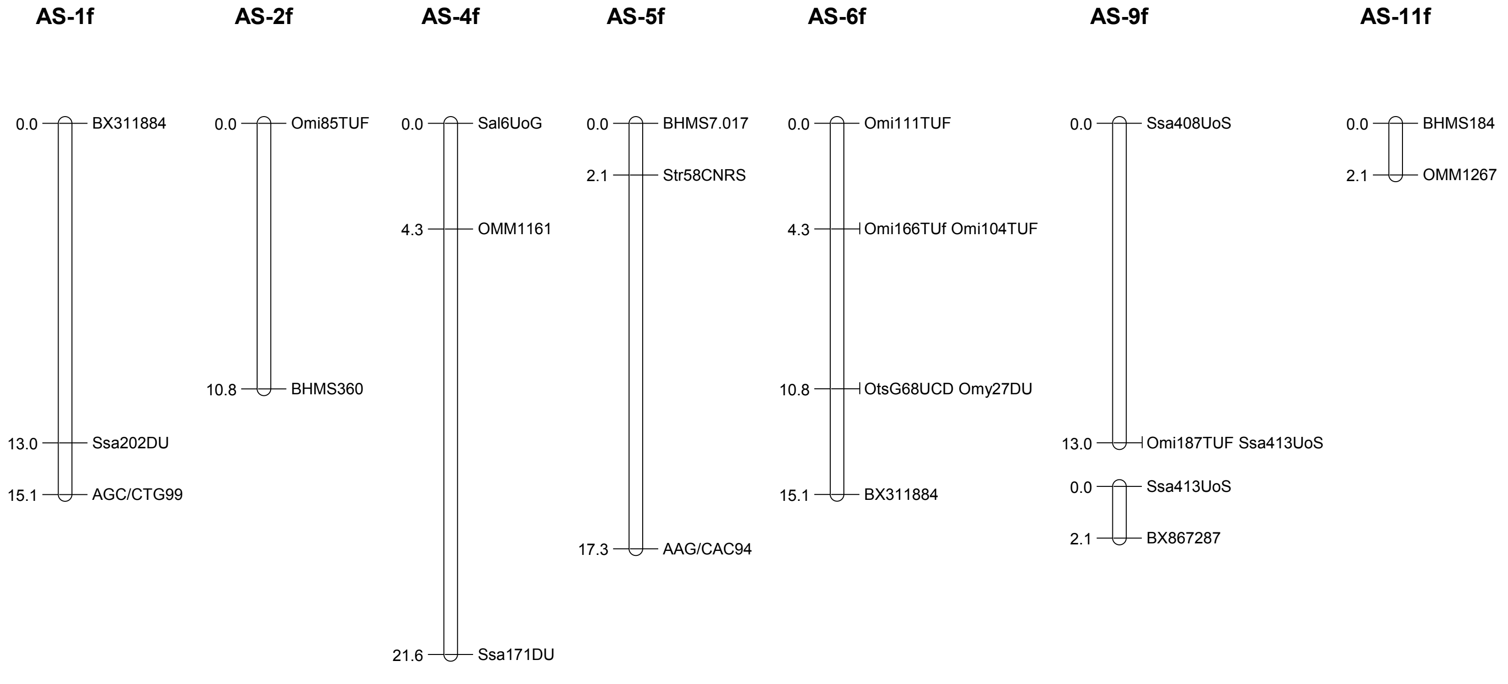

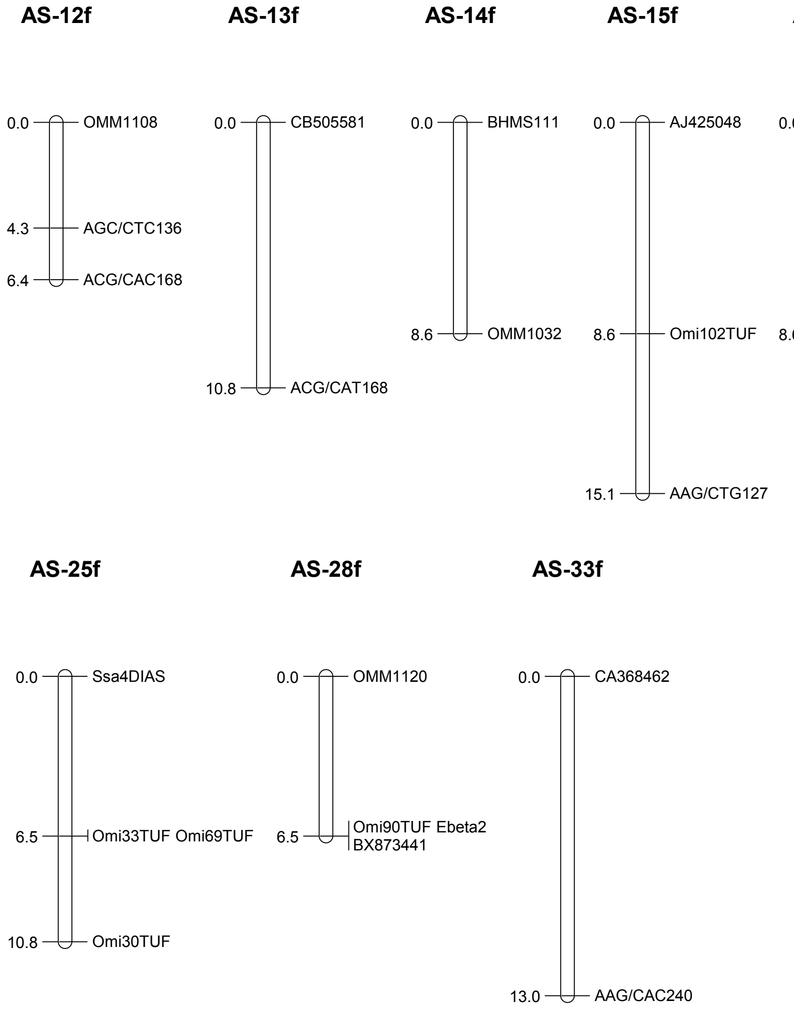

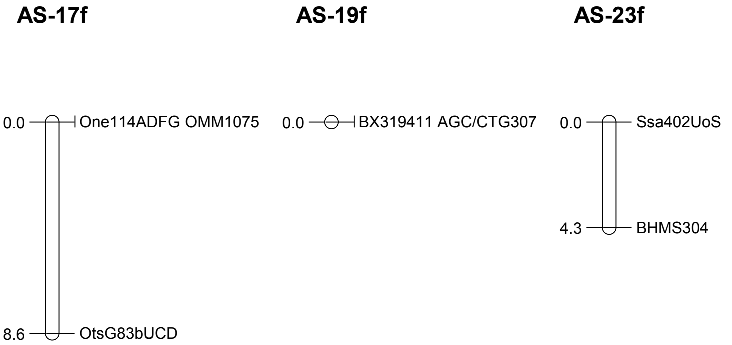

Linkage group designations for unlinked markers

Linkage Group	Marker
AS-1f	OMM1016
AS-4f	OMM1105
AS-4/10f	OmyRGT30TUF
AS-7f	BHMS103
AS-7f	BHMS117
AS-10f	One104ADFG
AS-11f	OmyRGT32TUF
AS-12f	BHMS267
AS-12f	OMM1189
AS-18f	BHMS420
AS-20f	BHMS241
AS-23f	Ssa20.19NUIG
