# Supplementary figures and images for: Genomic arrangement of salinity tolerance QTLs in salmonids: A comparative analysis of Atlantic salmon (Salmo salar) with Arctic charr (Salvelinus alpinus) and rainbow trout (Oncorhynchus mykiss)
Source: BMC Genomics. 2012 Aug 24;13:420. doi: 10.1186/1471-2164-13-420 (PMC3480877; doi:10.1186/1471-2164-13-420)

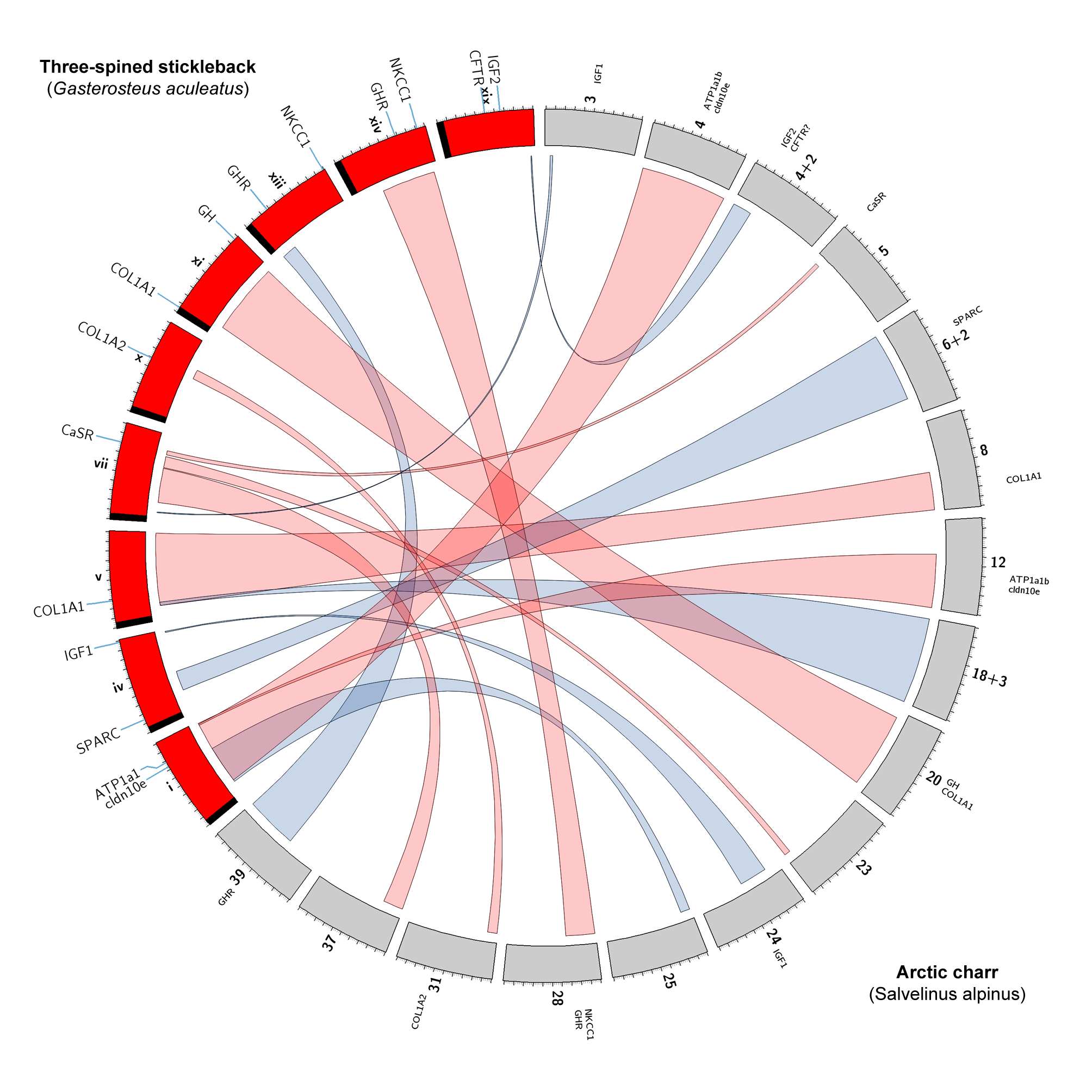

Supplement: Additional file 9 — Candidate gene predictions in Arctic charr (Salvelinus alpinus) based on synteny with three-spined stickleback (Gasterosteus aculeatus). [file 1471-2164-13-420-S9.png]

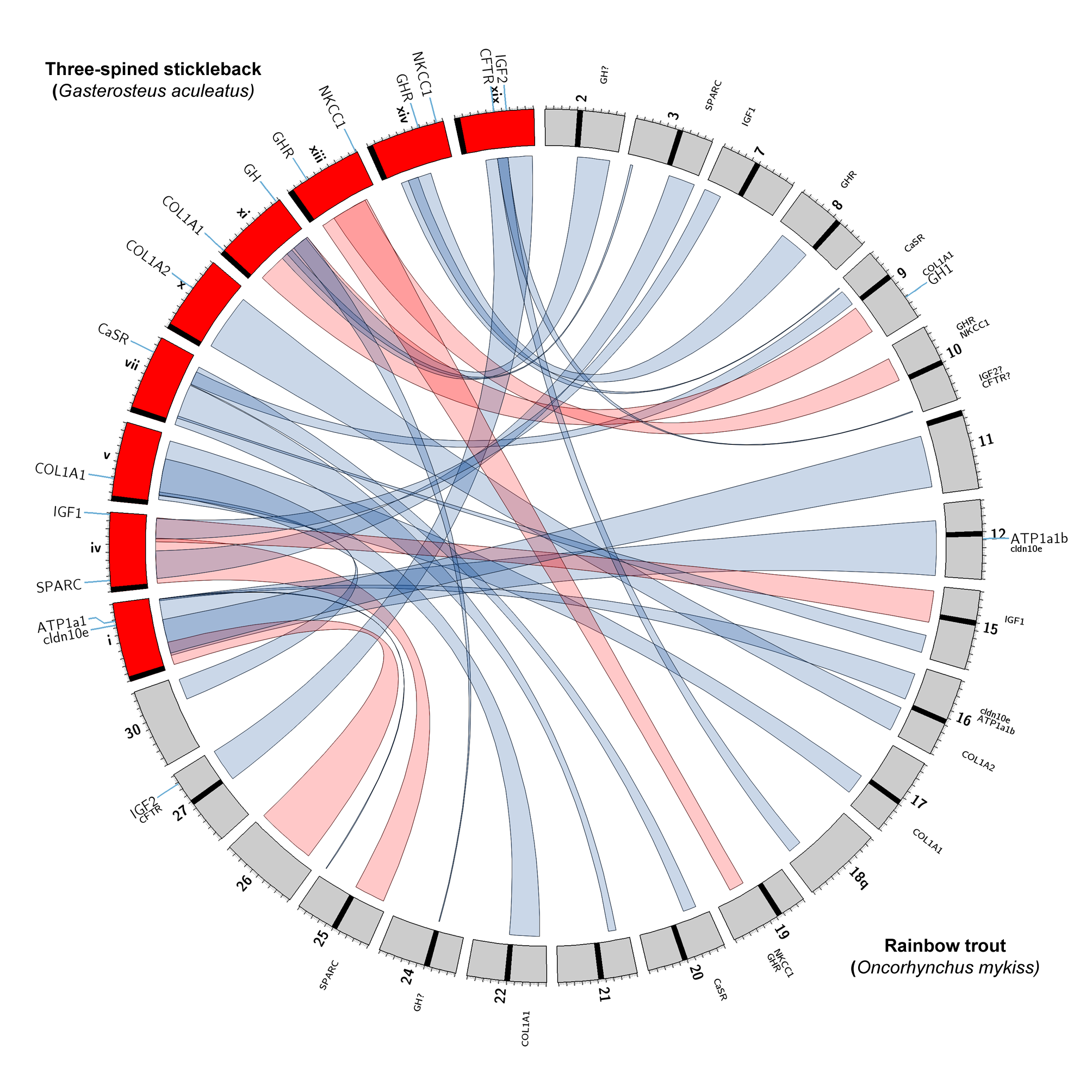

Supplement: Additional file 10 — Candidate gene predictions in rainbow trout (Oncorhynchus mykiss) based on synteny with three-spined stickleback (Gasterosteus aculeatus). [file 1471-2164-13-420-S10.png]
